# The anxiolytic effects of preoperative administration of pregabalin in comparison to diazepam and placebo

**DOI:** 10.1038/s41598-023-36616-0

**Published:** 2023-06-15

**Authors:** Sasikaan Nimmaanrat, Benjalak Charuenporn, Mark P. Jensen, Alan F. Geater, Jutarat Tanasansuttiporn, Thavat Chanchayanon

**Affiliations:** 1 Department of Anesthesiology, Faculty of Medicine, Prince of Songkla University, Hat Yai, Songkhla 90110 Thailand; 2 Department of Rehabilitation Medicine, University of Washington, Seattle, WA USA; 3 Department of Epidemiology, Faculty of Medicine, Prince of Songkla University, Hat Yai, Songkhla 90110 Thailand

**Keywords:** Health care, Medical research

## Abstract

We aimed to evaluate the potential anxiolytic effects of premedication with pregabalin, compared with diazepam and placebo. We conducted this non-inferiority, double-blind, randomized controlled trial in ASA classification I-II patients aged 18–70 years, scheduled for elective surgery under general anesthesia. They were allocated to receive pregabalin (75 mg the night before surgery and 150 mg 2 h before surgery), diazepam (5 and 10 mg in the same manner) or placebo. Preoperative anxiety was evaluated using verbal numerical rating scale (VNRS) and Amsterdam Preoperative Anxiety and Information Scale (APAIS) before and after premedication. Sleep quality, sedation level, and adverse effects were assessed as secondary outcomes. A total of 231 patients were screened and 224 completed the trial. The mean change (95%CI) in anxiety scores from before to after medication in pregabalin, diazepam, and placebo groups for VNRS were − 0.87 (− 1.43, − 0.30), − 1.17 (− 1.74, − 0.60), and − 0.99 (− 1.56, − 0.41), and for APAIS were − 0.38 (− 1.04, 0.28), − 0.83 (− 1.49, − 0.16), and − 0.27 (− 0.95, 0.40). The difference in change for pregabalin versus diazepam was 0.30 (− 0.50, 1.11) for VNRS and 0.45 (− 0.49, 1.38) for APAIS, exceeding the limit of inferiority for APAIS of 1.3. Sleep quality was statistically different between pregabalin and placebo groups (*p* = 0.048). Sedation in pregabalin and diazepam groups were significantly higher than placebo group (*p* = 0.008). No significant differences of other side effects, except dry mouth was higher in placebo group compared with diazepam (*p* = 0.006). The study filed to provide evidence at non-inferiority of pregabalin compared to diazepam. Furthermore, premedication with either pregabalin or diazepam did not significantly reduce the preoperative anxiety in comparison to placebo, despite the fact that both resulted in higher levels of sedation. Clinicians should weigh the benefits and risks of premedication with these 2 drugs.

Thai Clinical Trials Registry: TCTR20190424001 (24/04/2019) Registry URL: https://www.thaiclinicaltrials.org/.

## Introduction

Preoperative anxiety is common and remains an important problem for individuals scheduled for major surgery^1,2^. It has been reported to be the most distressing response to surgery^3^, and is associated with postoperative pain^4^, morbidity^5^, and mortality^5,6^.

Premedication with benzodiazepines has been in used for decades to ease anxiety and promote sleep in patients scheduled for surgery^7^. Benzodiazepines have both anxiolytic and amnestic effects. They exert their action as positive allosteric modulators on gamma amino butyric acid (GABA)-A receptor, which is a ligand-gated chloride-selective ion channel. GABA is the most common neurotransmitter in the central nervous system, and has high concentrations in limbic system and cortex. GABA has an inhibitory function, and thus decreases neurons’ excitability resulting in a calming effect^8^. However, it has been demonstrated that premedication with lorazepam in patients undergoing general anesthesia was related to modestly extended time to extubation and a lower rate of early recovery of cognition^9^.

Pregabalin (S-[+]-3-isobutylgaba) is a lipophilic GABA analog^10^. However, it does not bind to GABA-A, GABA-B or benzodiazepine receptors. It is not metabolized to GABA or a GABA agonist, nor does it have any influence on the uptake or degradation of GABA^11^. The primary mechanism of action of pregabalin involves inhibition of depolarization-induced calcium influx at P, Q and N-type voltage-gated calcium channels, leading to reduced release of excitatory neurotransmitters such as glutamate from nerve terminals. Its principal site of action is α2δ subunit of presynaptic, voltage-dependent calcium channels which are widely distributed throughout central and peripheral nervous system^12,13^. It is an anticonvulsant used for treating partial epilepsy^14^, neuropathic pain^15,16^ and generalized anxiety disorder (GAD)^17,18^.

Pregabalin is also used as a sleep-modulating drug^19^. A recent systematic review on preoperative administration of pregabalin for controlling anxiety which included 12 high quality trials has found that preoperative pregabalin 75 mg reduces anxiety and stabilizes intraoperative hemodynamic status. However, pregabalin 150 mg administered at least 1 h prior to surgery appears to provide a greater control of anxiety without causing any additional serious adverse consequences^20^. For these reasons, pregabalin may be a viable alternative to diazepam for treating preoperative anxiety, especially for patients who might not be able to tolerate diazepam or otherwise would prefer to avoid taking a benzodiazepine.

Given these considerations, the primary aim of this double-blind randomized controlled trial was to compare the anxiolytic effects of premedication with pregabalin, diazepam or placebo in patients scheduled for elective surgery. We hypothesized that patients randomly assigned to receive either pregabalin and diazepam would endorse lower levels of anxiety post-treatment than patients assigned to placebo, but that patients assigned to receive pregabalin and diazepam would not evidence significantly different levels of post-treatment anxiety. The secondary aims were to compare the 3 treatment conditions to each other with respect to sleep quality, overall sedation and rates of adverse events.

## Methods

### Ethics

This study was approved by the Ethics Committee of the Faculty of Medicine, Prince of Songkla University, Thailand: REC Number 62-080-8-1 (19/07/2019) and registered with Thai Clinical Trials Registry TCTR20190424001 (24/04/2019). All participants provided written informed consent. Study recruitment began on 29 August, 2019 and continued through 19 December, 2019. The data was anonymized, maintained with confidentiality and in compliance with the Declaration of Helsinki.

### Study design and patient selection

This is a randomized, double-blind, placebo-controlled trial. In order to be eligible to participate, potential participants needed to be aged 18 to 70 years old, be scheduled for an elective surgery under general anesthesia, and be rated as having American Society of Anesthesiologists (ASA) physical status I or II.

Exclusion criteria included being unable to understand Thai language or provide a written informed consent, having a history of allergy to pregabalin or diazepam, having a history of using pregabalin or diazepam on a regular basis, being pregnant or breast feeding, having renal function impairment (CrCl < 60 mL/min/1.73 m^2^), having a current psychiatric disorder, epilepsy or obstructive sleep apnea, being morbid obesity (BMI > 35 kg/m^2^), being at risk for aspiration or difficult airway, and having a history of drug abuse.

### Randomization and procedure

Study participants were blindly and randomly assigned into one of 3 groups, using a computer-generated block of six randomization: pregabalin (group PGB), diazepam (group DZP) and placebo (group PBO). They were allocated to receive pregabalin (75 mg the night before surgery and 150 mg 2 h before surgery), diazepam (5 and 10 mg in the same manner) or placebo. (Details in Table 1) As it was shown in a systematic review^20^ that administration of pregabalin 150 mg at least 1 h prior to surgery led to reduction of anxiety and as we used to routinely premedicate our patients with diazepam 5 mg the night before surgery and 10 mg 1–2 h before surgery, we administered pregabalin in the same manner (75 mg the night before surgery and 150 mg 2 h before surgery) to our patients in this study.

**Table 1:** How patients were premedicated with study medications.

Allocation	Night before surgery	Two hours before surgery
Group PGB	1 capsule of pregabalin (75 mg) 1 diazepam capsule filled with starch	2 capsules of pregabalin (75 mg) 2 diazepam capsules filled with starch
Group DZP	1 capsule of diazepam (5 mg) 1 pregabalin capsule filled with starch	2 capsules of diazepam (5 mg) 2 pregabalin capsules filled with starch
Group PBO	1 pregabalin capsule filled with starch 1 diazepam capsule filled with starch	2 pregabalin capsules filled with starch 2 diazepam capsules filled with starch

*PGB* pregabalin, *DZP* diazepam, *PBO* placebo.

Placebo capsules were prepared by filling starch into the emptied pregabalin or diazepam capsules. Study medications were prepared and put into coded envelopes according to the allocation orders by an independent pharmacist who was not involved in data collection or analysis. All treating clinicians including the anesthesiologists and surgeons, as well as the study participants were blind to allocation. The study participants were not prescribed any other medications at presurgery.

On the day before surgery, demographic data, baseline vital signs and preoperative anxiety were recorded by BC, who was blind to treatment assignment. At the preoperative holding area, after all participants had premedication, preoperative anxiety was reassessed by BC. Sleep quality for the previous night, sedation scores and drug side effects were also evaluated at this time. See measures section below for a description of all outcome measures. Administration of general anesthesia was performed by the attending anesthesiologists. After surgery, the study participants were taken to the postanesthesia care unit (PACU) for observation.

### Measures

#### Descriptive measures

Demographic data (sex, age, weight, height, body mass index (BMI), ASA classification and history of previous surgery), baseline clinical variables (vital signs and preoperative pain), and postoperative clinical variables (vital signs) were recorded.

#### Primary outcomes

We evaluated anxiety before and after receiving 2 doses the study medications prior to surgery using 2 co-primary outcome measures: (1) an 11-point Verbal Numerical Rating Scale (VNRS) with 0 = “Very calm” and 10 = “Very anxious”; and (2) the Amsterdam Preoperative Anxiety and Information Scale (APAIS) Thai version^21^. The original APAIS was developed in 1996 by the Dutch group of Moerman. It is a 6-item questionnaire which each item is rated on a 5 point Likert scale ranging from 1 “Not at all” to 5 “Extremely”. Four items ask about anxiety related to the anesthesia and surgery, and 2 items assess a perceived need for information. Evidence supports the validity and reliability of the APAIS for evaluating preoperative anxiety and perceived need for information^22^. In this study, only the 4 items assessing anxiety were used. Thus, the total score could potentially range from 4 to 20. In the current sample, the internal consistencies (Cronbach’s alphas) of the APAIS items before and after medication were 0.83 and 0.85, indicating good reliability.

#### Secondary outcomes

We assessed preoperative anxiety before receiving the study medications by State Trait Anxiety Inventory (STAI-state) form Y Thai version^21^. In addition, while the participants were in an operating room’s holding area, the previous night’s sleep quality was rated using a 0–10 VNRS with 0 = “Unable to sleep due to anxiety” and “10 = Deep and satisfactory sleep.” Preoperative current pain severity was measured by a VNRS, with 0 = “No pain” and 10 = “Worst pain imaginable.” Sedation at this time was rated by the experimenter using a standard 4-point Likert scale, with 0 = “Fully awake”, 1 = “Mild sedation—easy to rouse”, 2 = “Moderate sedation, easy to rouse but unable to remain awake”, and 3 = “Difficult to rouse”^23^.

### Study population size

This is a non-inferiority trial which hypothesized that the difference in the APAIS scores between participants who received pregabalin and diazepam would be 0. The standard deviation of the APAIS reported in a previous study with a proportional comparison of a visual analog scale of 2.6 was used^24^. Given 5% significance level, 90% power, and non-inferiority limit of 1.3, the minimum number of patients required in each group was 69. With an expected 10% dropout rate, the required sample size for study enrolment was 77 participants in each group, or 231 overall.

### Statistical analysis

We first computed descriptive statistics to describe the sample and compare the participants assigned to the 3 treatment conditions [using a Fisher’s exact test or Chi-square test as appropriate for the categorical variables, and analysis of variance (ANOVA) F-test or Kruskal–Wallis test as appropriate for the continuous variables] to determine if they were equivalent at baseline. Then, to test the primary hypothesis, we used a mixed-effects random-intercept linear regression with treatment condition as the primary independent variable and the APAIS and VNRS scores as the dependent variable. [Mean values of preoperative anxiety by the APAIS and VNRS (95% confidence interval) before and after receiving study medication in pregabalin, diazepam and placebo groups, the changes in preoperative anxiety by the APAIS and VNRS (95% confidence interval) from baseline and after receiving study medication in each group, and the differences in changes between groups (95% confidence interval) were analyzed using mixed-effects random-intercept linear regression to account appropriately for the repeated measures.] To examine the effects of treatment condition on the secondary outcome variables, we used Kruskal–Wallis and Rank Sum test to analyze sleep quality and Chi-square test for sedation level among the 3 groups. *P*-value less than 0.05 was considered as statistically significant. All statistical analyses were performed using R program version 3.1.1 (Vienna, Austria).

## Results

**Figure 1:**
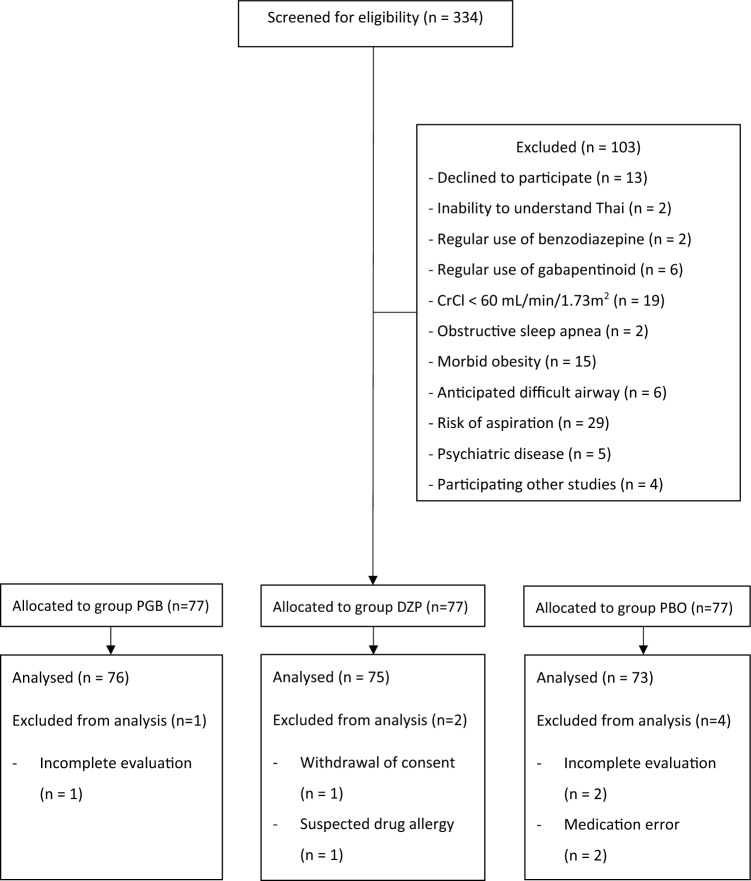
The CONSORT flowchart of this study. Abbreviations: CrCl, creatinine clearance; DZP, diazepam; PBO, placebo; PGB, pregabalin.

A total of 334 patients were screened for eligibility and 231 of these were enrolled. Seven patients were subsequently excluded, leaving 224 participants to complete the trial using an intention to treat analysis. (Fig. 1) As can be seen, there was no statistically significant differences between the participants in the 3 treatment conditions with respect to demographic data and the baseline measures of the primary and secondary outcomes. However, systolic blood pressure measured prior to induction of general anesthesia was statistically lower in groups PGB [135 (120,155) mmHg] and DZP [125 (115.5,150) mmHg] in comparison to group PBO [140 (128,160) mmHg], *p* = 0.004 (Table 2).

**Table 2:** Demographic data, baseline characteristics and vital signs after receiving premedication (n = 224).

Group	PGB (n = 76)	DZP (n = 75)	PBO (n = 73)	*P*-value
Demographic data				
Sex				
Female, n, %	56 (73.7)	63 (84)	55 (75.3)	0.265
Age (years), mean (SD)	46 ± 12.5	46.7 ± 10.5	46.5 ± 12.1	0.928
Weight (kg), mean (SD)	63.2 ± 11.4	60.5 ± 8.9	64.5 ± 10.2	0.054
Height (cm), median (IQR)	159 (153.9,165)	157 (152,163)	160 (154,165)	0.225
BMI (kg/m^2^), mean (SD)	24.9 ± 4	24.2 ± 3.3	25.3 ± 4	0.264
ASA Classification, n (%)				0.195
I	18 (23.7)	15 (20)	9 (12.3)	
II	58 (76.3)	60 (80)	64 (87.7)	
Previous surgery, n (%)				0.972
Yes	50 (65.8)	49 (65.3)	49 (67.1)	
No	26 (34.2)	26 (34.7)	24 (32.9)	
Baseline characteristics				
Vital signs, mean (SD)/median (IQR)				
Body temperature (°C)	36.8 (36.6,37)	36.8 (36.6,37)	36.8 (36.5,37.1)	0.979
Pulse rate (bpm^1^)	80.1 ± 13.1	79.8 ± 11.6	81.7 ± 13.2	0.630
Respiratory rate (bpm^2^)	20 (20,21.2)	20 (20,20)	20 (20,20)	0.920
Systolic BP (mmHg)	125.3 ± 15.1	127.9 ± 15.3	129 ± 16.6	0.334
Diastolic BP (mmHg)	75.4 ± 10	74.9 ± 10.1	76.3 ± 9.8	0.680
Preoperative pain measured by VNRS, median (IQR)	0 (0,0)	0 (0,2)	0 (0,2)	0.174
Baseline preoperative anxiety, median (IQR)				
VNRS	4 (2,5.25)	5 (2,6)	5 (3,7)	0.187
APAIS	7.5 (5,10.25)	8 (5.5,10)	8 (6,11)	0.611
STAI	29 (23,36)	31 (25.5,38.5)	34 (23,42)	0.297
Vital signs after premedication, mean (SD)/median (IQR)				
Body temperature (°C)	36.2 ± 0.6	36.2 ± 0.5	36.3 ± 0.4	0.535
Pulse rate (bpm^1^)	70 (62.8,85)	75 (65.5,84.5)	75 (65,90)	0.201
Respiratory rate (bpm^2^)	20 (16,20)	20 (16,20)	20 (18,20)	0.399
Systolic BP (mmHg)*	135 (120,155)^ab^	125 (115.5,150)^a^	140 (128,160)^b^	0.004
Diastolic BP (mmHg)	80 ± 12.2	77.2 ± 13.4	80.5 ± 12.3	0.224
Preoperative anxiety after receiving medications, median (IQR)				
VNRS	3 (0,5)	2 (1,5)	5 (0,5)	0.243
APAIS	8 (5,11)	6 (5,9)	8 (5,11)	0.456

*APAIS* Amsterdam preoperative anxiety and information scale, *ASA* American Society of Anesthesiologists, *BP* blood pressure, *bpm*^*1*^ beats per minute, *bpm*^*2*^ breaths per minute, *DZP* diazepam, *IQR* interquartile range, *PBO* placebo, *PGB* pregabalin, *SD* standard deviation, *STAI* Spielberger’s state-trait anxiety inventory, *VRNS* verbal numerical rating scale.

*Value not having a superscript in common, differs significantly (*P* < 0.05, Rank Sum test after significant Kruskal–Wallis test).

Baseline preoperative anxiety as measured by the VNRS [median (IQR)] in the PGB, DZP and PBO groups were 4 (2,5.25), 5 (2,6), and 5(3,7), respectively. Preoperative anxiety after receiving study medications measured by the VNRS [median (IQR)] were 3 (0,5), 2 (1,5), and 5 (0,5), respectively. Neither the pre- nor post-medications scores were significantly different from one another (*p*’s = 0.187 and 0.243, respectively).

**Figure 2:**
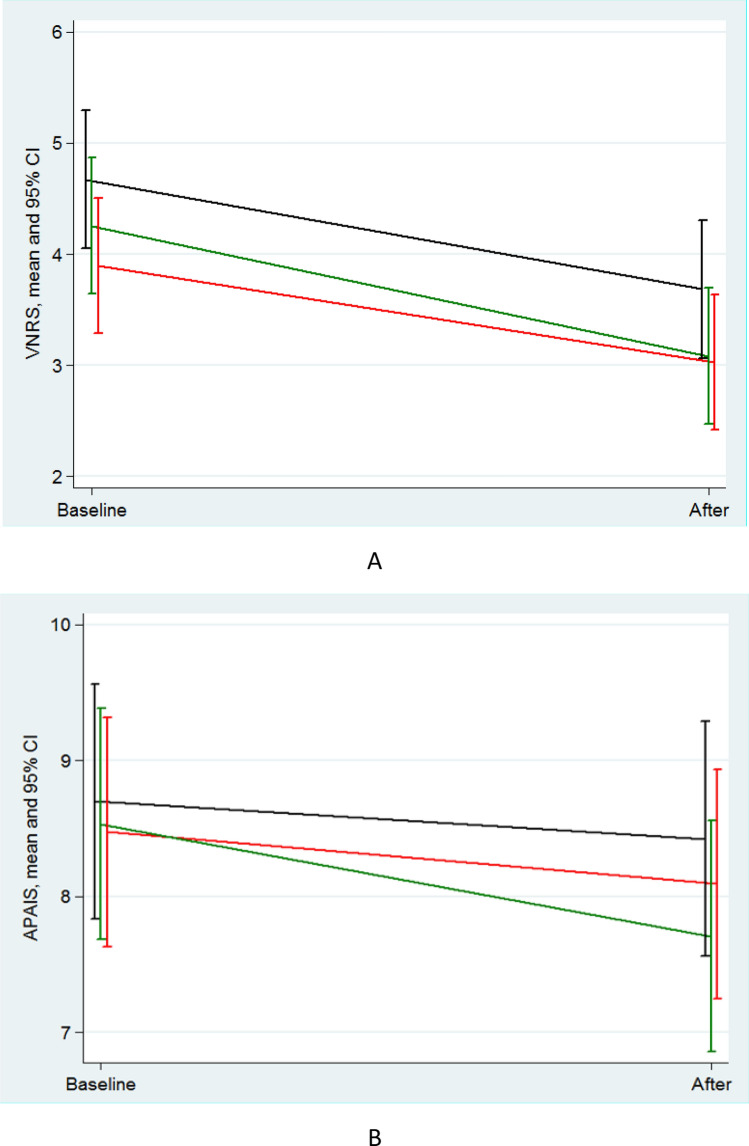
Margin graph of preoperative anxiety by (**A**). Verbal numerical rating scale (VNRS) and (**B**). Amsterdam preoperative anxiety and information scale (APAIS) at baseline and after receiving study medication from mixed-effects random-intercept linear regression model. Pregabalin (red), diazepam (green) and placebo (black).

The results of the mixed effects linear regression analyses revealed that the anxiety score measured by the VNRS decreased significantly from pre- to post-medication in all groups (including PBO group), with no significant difference in the reduction between PGB and DZP groups, or between either medication and PBO group (Table 3A, Fig. 2A).

**Table 3:** Preoperative anxiety by A. Verbal numerical rating scale (VNRS) and B. Amsterdam preoperative anxiety and information scale (APAIS) at baseline and after receiving study medication in pregabalin, diazepam and placebo group.

Group	Change in preoperative anxiety: VNRS (After—Baseline)	Difference between the change in preoperative anxiety		
		(PGB-DZP)	(PGB-PBO)	(DZP-PBO)
A. Verbal numerical rating scale (VNRS)				
PGB	− 0.87 (− 1.43 to − 0.30) (*p* = 0.003)	0.30 (− 0.50 to 1.11) (*p* = 0.457)	0.12 (− 0.69 to 0.93) (*p* = 0.775)	− 0.19 (− 1.00 to 0.62) (*p* = 0.651)
DZP	− 1.17 (− 1.74 to − 0.60) (*p* < 0.001)			
PBO	− 0.99 (− 1.56 to − 0.41) (*p* = 0.001)			

Group	Change in preoperative anxiety: APAIS (After—Baseline)	Difference between the change in preoperative anxiety		
		(PGB-DZP)	(PGB-PBO)	(DZP-PBO)
B. Amsterdam preoperative anxiety and information scale (APAIS)				
PGB	− 0.38 (− 1.04 to 0.28) (*p* = 0.257)	0.45 (− 0.49 to 1.38) (*p* = 0.352)	− 0.11 (− 1.05 to 0.84) (*p* = 0.823)	− 0.55 (− 1.50 to 0.39) (*p* = 0.253)
DZP	− 0.83 (− 1.49 to − 0.16) (*p* = 0.015)			
PBO	− 0.27 (− 0.95 to 0.40) (*p* = 0.426)			

Using mixed-effects random-intercept linear regression.

Data are presented as mean (95% confidence interval).

*PGB* pregabalin, *DZP* diazepam, *PBO* placebo.

The mean changes (95%CI) in anxiety score from before to after medication in PGB, DZP and PBO groups for VNRS were − 0.87 (− 1.43, − 0.30), − 1.17 (− 1.74, − 0.60), and − 0.99 (− 1.56, − 0.41). The difference in change in VNRS (PGB-DZP) was 0.30 (− 0.50, 1.11) (*p* = 0.457). There were no significant differences between the change from baseline between PBO group and either of the 2 medications (Table 3A, Fig. 2A).

The baseline preoperative anxiety measured by the APAIS [median (IQR)] in the PGB, DZP and PBO groups were 7.5 (5,10.25), 8 (5.5,10), and 8 (6,11), respectively. (*p* = 0.611) Preoperative anxiety after receiving study medications measured by the APAIS [median (IQR)] were 8 (5,11), 6 (5,9), 8 (5,11), respectively. (*p* = 0.456) (Table 3B, Fig. 2B).

The mean changes (95% CI) in anxiety score from before to after medication in PGB, DZP and PBO groups for APAIS were − 0.38 (− 1.04, 0.28), − 0.83 (− 1.49, − 0.16), and − 0.27 (− 0.95, 0.40). There were no significant differences between the change from baseline between PBO group and either of the other 2 medication groups (Table 3B, Fig. 2B).

The difference between the change in preoperative anxiety measured by the APAIS in the PGB group and DZP group (PGB-DZP) was 0.45, with a 95% confidence interval of − 0.49 to 1.38. As this confidence limit exceeds the limit of non-inferiority (the non-inferiority limit of 1.3 as indicated in the sample size calculation section), the study failed to provide evidence of the non-inferiority of pregabalin compared with diazepam (Table 3B).

The sleep quality assessed by the VNRS [median (IQR)] after receiving the night dose of study medications, demonstrated a statistically significant difference between the PGB group and PBO group. However, the median value was the same in all 3 groups (Table 4).

**Table 4:** Sleep quality assessed by the VNRS after receiving the night dose of the study medications.

Group	PGB	DZP	PBO	*P*-value
Sleep quality by VNRS	8 (7,10)^a^	8 (6,10)^ab^	8 (6,9)^b^	0.048

Data are presented as median (IQR).

Value not having a superscript in common, differs significantly (*P* < 0.05, Rank Sum test after significant Kruskal–Wallis test).

There were no significant between-group differences in the rates of most side effects including dizziness, peripheral edema, blurred vision, thinking abnormally or postoperative nausea and vomiting. However, the incidence of dry mouth was found to be higher in the PBO group (n = 26, 35.6%) than the PGB (n = 16, 21.1%) and DZP (n = 11, 14.7%) groups. The between-group difference was statistically significant only between the PBO and DZP group (*P* = 0.006).

Finally, the level of sedation was statistically significantly higher in the PGB and DZP groups compared with the PBO group (*P* = 0.008). The incidence of sedation score of at least 2 in the PGB, DZP and PBO groups were 9.2%, 13.3% and 4.1%, respectively. (Table 5) After surgery, 1 patient in the DZP group had excessive sedation needing prolonged observation in PACU. However, no serious respiratory depression or hemodynamic instability occurred.

**Table 5:** Sedation score prior to surgery.

Sedation score	Group PGB^a^ (n = 76) n (%)	Group DZP^a^ (n = 75) n (%)	Group PBO^b^ (n = 73) n(%)	*P*-value
0	39 (51.3)	38 (50.7)	55 (75.3)	0.008
1	30 (39.5)	27 (36)	15 (20.5)	
2	7 (9.2)	7 (9.3)	3 (4.1)	
3	0 (0)	3 (4)	0 (0)	

Data are presented as number (%).

Value not having a superscript in common, differs significantly (*P* < 0.05, Chi Square test).

*DZP* diazepam, *PBO* placebo, *PGB* pregabalin.

Sedation score: 0, no sedation—fully awake; 1, easy to rouse; 2, easy to rouse but cannot stay awake; 3, difficult to rouse.

## Discussion

The purpose of this study was to determine if patients premedicated with pregabalin would report similar improvements (decreases) in anxiety as those who received the standard treatment, diazepam, for anxiety in this population. The findings indicated that the patients who received the 2 medications evidenced similar improvements in anxiety overtime. However, and unexpectedly, we also found that those who received placebo evidenced deceases in anxiety, and that the anxiety decreases associated with the active treatments were not significantly greater than those that occurred with placebo treatment.

Preoperative anxiety is common. The prevalence of clinically meaningful preoperative anxiety has been shown to vary from 12.6 to 59.6%^25–27^, depending on the population studied and the measures used to assess anxiety. A systematic review and meta-analysis has concluded that the global prevalence of preoperative anxiety among patients scheduled for various kinds of surgical procedures is still very high at almost 50%. Preoperative anxiety has been shown to be approximately 4 times higher in patients with a fear of complications^28^. In addition, pregabalin has been shown to be consistently effective for the treatment of generalized anxiety disorder (GAD)^29^. Consistent with these findings, in a recent critical review, 7 of 8 randomized controlled trials in generalized anxiety disorder revealed a statistically significant effect of pregabalin 150–600 mg on Hamilton Anxiety Rating Scale scores^30^.

In this study, we utilized 3 tools, the VNRS, APAIS and STAI, to measure preoperative baseline anxiety level before receiving any medications. After the patients had received both the night and on the day of surgery doses of study medications, we employed the VNRS and APAIS to assess the effects of these medications on preoperative anxiety level. All of our patients experienced preoperative anxiety. Our study has shown no significant reduction in preoperative anxiety after receiving pregabalin, diazepam or placebo. This finding is consistent with the results of a systematic review conducted in 2015, which found that the role of pregabalin in reduction of preoperative anxiety measured by visual analog scale in elective surgery remained inconclusive^31^. Our findings are also consistent with those from a dose ranging study of pregabalin, which found that premedication with pregabalin in elective ambulatory and short-stay surgical patients failed to lessen anxiety level in all dose ranges (75–300 mg)^32^.

Studies conducted by Fassoulaki et al.^33^ and Gurunathan et al.^34^ also found no significant difference in anxiety score between patients who received pregabalin preoperatively and placebo or other study medications. However, these studies were powered to detect the effects of medications on opioid consumption within the first 24–48 h after the surgical procedures, and postoperative pain at rest and with coughing or movement. Thus sample sizes may not have been adequate for evaluating the anxiolytic effects of pregabalin.

At the same time, many studies have found a positive association between premedication with pregabalin and reducing preoperative anxiety. Gonano et al.^35^ administered a single dose of pregabalin 300 mg preoperatively to outpatients scheduled for minor orthopedic surgery, and found a significant reduction of preoperative anxiety up to 40%. However, it is important to point out that Gonano et al. used different dosing procedure than we did. We provided our participants with 2 administrations of 2 different doses of pregabalin in inpatients scheduled for various kinds of major surgery. Spreng et al.^24^ gave pregabalin 150 mg to patients undergoing discectomy, and found a significant reduction of preoperative anxiety score with this treatment. This result was consistent with another study by Shimony et al.^36^ in patients undergoing brain tumor removal who received pregabalin 150 mg on an evening and 1.5 h before surgery. Waikar et al.^37^ gave preoperative pregabalin 150 mg to patients planned for elective surgery under general anesthesia and found that it reduced anxiety significantly. A recent systematic review of 12 high quality randomized controlled trials concluded that preoperative administration of a single dose pregabalin 150 mg appears to be effective for reducing anxiety significantly^20^. For minor procedures performed under local anesthesia, Elrashidy et al.^38^ found that patients scheduled for vitreo-retinal surgery who received preoperative pregabalin demonstrated less anxiety in comparison to placebo. Eskandarian et al.^39^ premedicated 4–6 years old children scheduled for dental treatment with pregabalin 75 mg and found that it reduced anxiety score significantly.

The inconstant findings of the efficacy of pregabalin to reduce preoperative anxiety may be related to confounding factors such as different study population, doses and timing of pregabalin administration, as well as methods and timing of anxiety assessment.

Regarding comparison of anxiolytic effect between pregabalin and diazepam, one randomized control trial conducted by Jokela et al.^40^ compared premedication with pregabalin 300 or 600 mg and diazepam 10 mg, and reported a comparable level of anxiety after the administration of these medications, assessed before anesthetic induction in laparoscopic hysterectomy patients. However, this study did not evaluate the degree of reduction in preoperative anxiety because of its lack of baseline preoperative anxiety data.

Regarding sleep quality, the available data have showed that pregabalin has positive benefits on sleep quality. In rats, pregabalin has been shown to decrease the duration of rapid eye movement sleep (REMS) and significantly increased non rapid eye movement sleep (NREMS)^41^. In healthy adult volunteers, pregabalin has been shown to markedly augment slow-wave sleep as well as decreased sleep-onset latency and number of awakenings of more than 1 min in duration^19^. Pregabalin has also been shown to improve disturbed sleep parameters in patients with fibromyalgia by preventing awakenings and increasing sleep bout duration^42^. Our patients receiving pregabalin demonstrated better sleep quality in comparison to those receiving placebo, which is consistent with the previous trial by Shimony et al.^36^.

We also found that patients receiving either pregabalin or diazepam had higher sedation scores in comparison to placebo. This finding was consistent with previous studies^43–46^. However, there were no serious adverse events associated with either treatment, such as respiratory depression or hemodynamic disturbance occurred in either group.

This study aimed to compare the preoperative anxiolytic effect of pregabalin with an active comparator, diazepam as well as placebo. We incorporated 2–3 anxiety assessment tools for confirming patients’ preoperative anxiety level. This study has a number of limitations which should be considered when interpreting the findings. First, our study population consisted of patients scheduled for various kinds of elective surgical procedures as this might pose different influence on preoperative anxiety level. We might have found greater efficacy of one or both of the medications studied had we limited that sample to a single type of surgery or surgical procedure with higher anxiety. Our study participants underwent elective surgery so the results may not be valid to surgical patients with urgent or emergent indications. Also, it is important to note that oral pregabalin is rapidly and completely absorbed with peak level within 1 h and over 90% bioavailability^11^. Its onset of substantial anxiolytic effect is within 3–4 h after a single dose^45^. Although, our study participants received 2 doses of pregabalin, assessing anxiety at 2 h postdose on the day of operation might be too soon to detect its clinically meaningful anxiolytic action. Future studies should examine the effects of pregabalin when administered earlier.

Despite these limitations, the study provides information regarding the effects of both pregabalin and diazepam for reducing preoperative anxiety. In a fully powered study that included more subjects than have been included in prior studies, allowing for a direct head-to-head comparison of pregabalin with diazepam, and both of these medications with placebo, and using 2 valid measures of anxiety, we found that in the population studied, while the 2 active treatments were associated with similar beneficial effects, neither evidenced larger effects on preoperative anxiety than placebo. Their findings suggest the possibility that neither treatment may be effective for reducing anxiety for all patients undergoing surgery. More research is therefore needed to help determine the specific populations that might be most responsive to either or both treatments.

## Conclusions

The study did not provide evidence at non-inferiority of pregabalin compared to diazepam. However, both of them failed to demonstrate anxiolytic effect in comparison to placebo despite the fact that they increased level of sedation. This would suggest that pregabalin and diazepam are not an anxiolytic of choice for premedication in patients scheduled for elective surgery.

## Data Availability

The datasets used and/or analyzed during the current study are available from the corresponding author on reasonable request.
